# Out of hours care in Germany - High utilization by adult patients with minor ailments?

**DOI:** 10.1186/s12875-017-0609-1

**Published:** 2017-03-21

**Authors:** R. Leutgeb, P. Engeser, S. Berger, J. Szecsenyi, G. Laux

**Affiliations:** University Hospital Heidelberg, Marsilius-Arcades, Western Tower, Im Neuenheimer Feld 130.3, 69120 Heidelberg, Germany

**Keywords:** Out-of-Hours Care (OOHC), Minor ailments, Low urgency, Patient perspective, Reason for Encounter (RFE)

## Abstract

**Background:**

Family practitioners (FPs) who work in Out-Of-Hours Care (OOHC) - especially in rural areas – complain about high workload related to low urgency and potentially unnecessary patient presentations with minor ailments. The aim of this study was to describe Reasons for Encounter (RFEs) in primary OOHC taken into account the doctor’s perspective in the context of high workload without knowing patients’ motives for visiting an OOHC-centre.

**Methods:**

Within this descriptive study, OOHC data from 2012 were evaluated from a German statutory health insurance company in the federal state of Baden-Wuerttemberg. 1.53 Million of the 10.5 Million inhabitants of Baden-Wuerttemberg were covered. The frequency of the ICD-10 diagnoses was determined at the three- and four-digit-level. The rate of hospitalizations was used to estimate the severity of the evaluated cases.

**Results:**

Taken as a whole, 163,711 reasons for encounter with 1,174 ICD-10 single diagnoses were documented, of these 62.2% were on weekends. Less than 5.0% of the examined patients were hospitalized. Low back pain-dorsalgia (M54) was the most common diagnosis in OOHC, with 10,843 cases. Injuries were found twelve times in the list of the 30 most frequent diagnoses. The most frequent infectious disease was acute upper respiratory infection of multiple and unspecified sites (J06). By analysing the ICD codes to four-digits and looking at the rate of hospitalizations, it can be assumed that many RFEs were of less urgency in terms of the prompt need for medical treatment.

**Conclusion:**

While it is acknowledged that it can be difficult to make an exact diagnosis in an OOHC setting, after analysing the ICD-10 diagnoses, the majority of reasons for encounter in OOHC were determined to be of low urgency, meaning that patients could have waited until regular consultation hours. In the OOHC setting, it is important to understand RFEs from both the patient perspective and the family practitioner perspective. Additionally, results like these can be used in staff education especially improving triage methods and medical recommendations and in developing specific guidelines for OOHC in Germany. Analysis of routine data, such as in this study, contributes to this understanding and contributes to resolving problems of coding.

**Electronic supplementary material:**

The online version of this article (doi:10.1186/s12875-017-0609-1) contains supplementary material, which is available to authorized users.

## Background

In the last two decades in many Western countries, reforms in Out-Of-Hours Care (OOHC) have been initiated, partially, as a consequence of changing attitudes among Family Practitioners (FPs) [1, 2]. Patients presenting with minor ailments and the shortage of FPs in rural areas has intensified reform efforts [3–5]. In Germany, OOHC reforms initiated by the associations of statutory health insurance physicians of the different federal republics of Germany commenced in 2014. In Hesse and Baden Wuerttemberg, two of these federal states, OOHC districts were enlarged in order to distribute the OOHC duties to more physicians in one region.

In addition, new OOHC-centres were founded, if possible near hospitals, and consistent remuneration for the physicians on duty was agreed in consensus. Costs for these services are now distributed to all physicians regardless of whether they practice in urban or rural regions [6].

In the context of these reforms, one important question in OOHC, discussed by physicians and health services researchers in many European countries, is how to identify patients with urgent health care problems in OOHC and, on the other hand, how to distinguish them from patients with minor ailments triaged as being safe to treat the following day in regular family practice care. One solution are triage systems, like the Manchester Triage System (MTS), the National Guide of the Dutch College of General Practitioners (NTG), which are applied in emergency departments or in general practice OOHC-centres in the Netherlands and other countries [7–11]. As a positive side-effect of triaging, screening of incoming calls from patients with potentially minor ailments that could be advised by phone might be identified. Campbell et al. showed that telephone triage in primary care might be an improvement for FPs’ daily work. But telephone triage in regular care means potentially more primary care contacts and OOHC-contacts after introducing such an option because telephone triage is an estimation of the severity of an ailment without face-to-face-contact [12–14]. In the UK and Canada, OOHC-reforms were launched to include pharmacists. Patients with “Minor Ailments”- defined by an expert-panel could be advised by pharmacists. This has good potential to reduce the work of overloaded physicians working in OOHC [15–17].

To date, there is no satisfactory widely accepted definition of the term “Minor Ailment”. One definition is that a “Minor Ailment” should be a self-limiting condition, managed by the patients themselves. In such cases, neither pharmacists nor FPs would need to intervene [18, 19]. However, a “Minor Ailment” has also been defined as an illness, but where a medical consultation could wait until the next day. This is the definition of the Netherlands Triage System (NTS), for urgency level 5 [7]. The triage categories in the MTS are nearly the same [10]. Additionally, we have to consider that all these definitions are developed by experts.

Within the given context the doctor’s on duty (FP) perspective is influenced by the special setting of fast decision making with limited diagnostic possibilities and handling a complex ICD-10 classification in the context of high workload. The patient’s points of view, their sociodemographic data and their feelings regarding the severity of their reasons for encounter [20–22], which can differ significantly from a medical opinion on the matter, have to be considered, too

The intention of this study was not to clarify the definition of minor ailments, but to analyze reasons for encounter in OOHC in a large sample and to list the frequency of ICD-10 diagnoses. On the basis of the four-digit level and on the basis of the rates of referrals to hospital the urgency of reasons for encounter was estimated.

## Methods

This descriptive study evaluated data, from 2012, supplied by a large German statutory health insurance company (AOK, “Allgemeine Ortskrankenkasse”) within the federal state of Baden-Wuerttemberg. The eligible study population consisted of 1.53 million adult insured individuals. The data was derived from a comprehensive evaluation data set of adult patients with state health insurance with AOK in Baden-Wuerttemberg. The *Hausarztzentrierte Versorgung* (HZV) (loosely translated as “family doctor coordinated care”) is a programme encouraging patients to enrol with a family doctor pursuant to Section 73b, Volume V of the German Social Security Code. It came into effect in Baden-Wuerttemberg, Germany, on July 1st, 2008. The HZV is aimed at enhancing health care for patients with chronic diseases and complex health care needs (e. g., those requiring long term care). This complex intervention, which is voluntary for both family doctors and patients, aims to strengthen the coordinative function of family practitioners. As a result, this intervention is believed to increase the quality of medical health care for insured individuals and thereby, ideally, to additionally save expenses. The details of this intervention are described elsewhere [23]. However, for this study we did not focus on the HZV intervention, but on all patients with OOHC utilization.

Originally, data were made available to the Department of General Practice and Health Services Research, University Hospital Heidelberg in order to assess the effectiveness of the HZV programme [23]. The AOK granted additional permission for data analysis for this study. Within the data set a specific reimbursement code - particular accounting digits for OOHC services - was used to unambiguously identify health care utilization in OOHC-centres. Reimbursement codes were only matched with single diagnoses to ensure a clear assignment of reasons for encounter in OOHC. Therefore, one third of diagnoses could not be used for an unambiguous determination of a reason for encounter (RFE).

The frequency of the ICD-10 diagnoses was determined at the three- and four-digits level.

Whenever the three-digit level was not specific enough, the four-digit-level was used. Data on referrals to hospital were also available in the data set and the rate of referrals was used additionally to estimate urgency of the need for medical treatment for specific cases. As data for the OOHC encounter included an exact date, a distinction between visits on workdays and weekend days was possible.

An expert panel of family doctors from the Department of General Practice and Health Services Research assessed urgency in terms of the need for medical treatment by means of the four-digit levels. Two GPs with long experience working in large family practices and coincidently being lecturers at the department of General Practice and Health Services Research, University Hospital Heidelberg, and two professors for health services research of the mentioned department, also with long experience, evaluated the ICD-10 diagnose codes in OOHC and in regular care.

In order to calculate frequencies, rates and percentages we used SAS PROC SQL (SAS 9.4 x64, SAS Institute Inc., Cary, NC, USA).

## Results

Two hundred sixty-three thousand two hundred sixty-one visits with 163,711 reasons for encounter, clearly matched to OOHC were evaluated for the 2012 period. 1,174 ICD-10 codes were given by the FPs on duty. Three hundred of the detected OOHC-ICD diagnoses covered nearly 90% of all RFEs. 62.2% of these RFEs accumulated on weekends.

Nearly 57% of the OOHC-patients were female. 32.8% of the OOHC-patients fell in to the age group between 18 and 39 years, 32% into the age group between 40 and 59 years, 25.5% in to the age group between 60 and 79 years and nearly 10% of the patients were at least 80 years old. Male patients (8.7% referral rate to hospital) and patients of higher age groups (nearly 10% referral rate to hospital) were admitted more frequently to hospital. The highest rates of referrals to hospital occurred on Saturday and Sunday (9.3% respectively 10.0%) (Table 1).

**Table 1:** Description of patient visits

Characteristic	Values		
Visits and referrals to hospital per gender	Age Group (Years)	Visits (n, %)	Ref. to Hospital (%)
	female	93,187 (56.9%)	5.2%
	male	70,524 (43.1%)	8.7%
Visits and referrals to hospital per age group	Age Group (Years)	Visits (n, %)	Ref. to Hospital (%)
	18–29	31,054 (19.0%)	5.2%
	30–39	22,577 (13.8%)	5.3%
	40–49	27,422 (16.8%)	5.5%
	50–59	24,830 (15.2%)	8.2%
	60–69	18,842 (11.5%)	8.5%
	70–79	22,976 (14.0%)	9.8%
	80–89	13,395 (8.2%)	9.7%
	≥90	2,615 (1.6%)	9.7%
Visits and referrals to hospital per day of week	Day of Week	Visits (n, %)	Ref. to Hospital (%)
	Monday	18,030 (11.0%)	6.6%
	Tuesday	14,031 (8.6%)	6.5%
	Wednesday	15,916 (9.7%)	7.3%
	Thursday	13,914 (8.5%)	6.2%
	Friday	18,781 (11.5%)	5.8%
	Saturday	44,514 (27.2%)	9.3%
	Sunday	38,525 (23.5%)	10.0%

We could observe that in the four subgroups -age (<= 50a and >50a) and gender (male/female) - the RFE ranking was very similar. For these subgroups Rank 1 was M54 (“Dorsalgia”) and Rank 2 was T14 (“Injury of unspecified body regions”). Moreover, J06 (Acute upper respiratory infections of multiple and unspecified sites), A09 (Diarrhea and other gastroenteritis and colitis of infectious and unspecified origin) and H10 (Conjunctivitis) were ranked within the “TOP 20” for all subgroups. The ten most frequent diagnoses, independent of age and gender, with referrals to hospital and utilization on weekends are listed in Table 2.

**Table 2:** Top ten most frequent diagnoses, referrals and utilization on weekends

Rank	ICD-10-Code	ICD-10-Text	n	Referrals to hospital in %	On weekend in %
1	M54	Dorsalgia	10,843	1.3	67.9
2	T14	Injury of unspecified body regions	7,104	2.6	68.2
3	S61	Open wound of wrist and hand	4,668	0.8	56.1
4	S93	Dislocation, sprain and strain of joints and ligaments at ankle and foot level	4,082	1.05	59.9
5	R10	Abdominal and pelvic pain	3,898	10.6	55.5
6	N39	Other disorders of urinary system	3,784	1.2	71.4
7	S01	Open wound of head	3,634	1.4	54.9
8	I10	Essential primaryhypertension	3,238	5.6	58.9
9	J06	Acute upper respiratory infections of multiple and unspecified sites	2,935	0.8	72.9
10	A09	Diarrhea and other gastroenteritis and colitis of infectious and unspecified origin	2,558	2.4	64.6

**Fig. 1:**
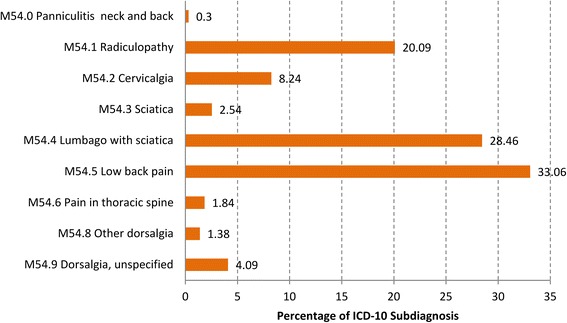
Distribution of the diagnosis “Low back pain-Dorsalgia” (M54) at the four-digit level

The most frequent diagnosis was “Dorsalgia–Low Back Pain” (M54), with 10,843 cases. From a medical point of view less severe diseases were documented at the four-digit level (Fig. 1).

**Fig. 2:**
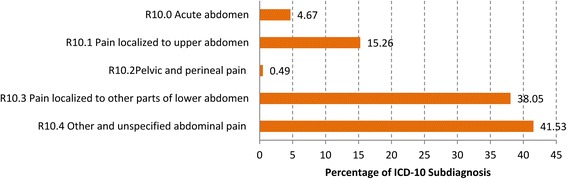
Distribution of the diagnoses “Injury of unspecified body region” (T14) at the four-digit level

Injuries were found twelve times within the most frequent 30 diagnoses, possibly sporting injuries. 59.2% of these injuries were seen on weekends (Fig. 2).

The most frequent infectious disease was the “Acute upper respiratory infection of multiple and unspecified sites” (J06) on rank position nine. Urinary tract infections were identified often, but not clearly coded e.g. classified by code N39 “Other Disorders of Urinary System”. The analysis of the referral rates to hospital of the ten most frequent diagnoses shows an overall rate of 2.5%. Two exceptions were in particular the diagnoses “Essential (primary) Hypertension” (I10) with a 5.6% rate of referrals to the hospital and the “Abdominal and Pelvic Pain” (R10) with a 10.6% rate of referrals (Table 2).

**Fig. 3:**
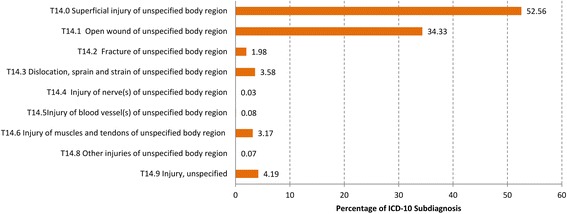
Distribution of the diagnoses “Essential (primary) hypertension” (I10) at the four-digit level

**Fig. 4:**
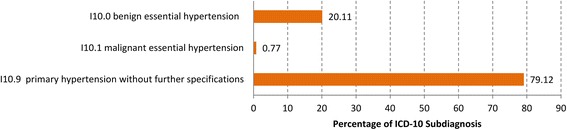
Distribution of the diagnoses “Abdominal and Pelvic Pain” (R10) at the four-digit level

If we look at the four-digit levels of these two ICD-10 codes we detect that only 0.8% of the ICD 10 diagnoses “Essential Hypertension” were coded with “Malignant Essential Hypertension”(I10.1) but 79.1% with the code “Essential Primary Hypertension without further Specifications”(I10.9), (Fig. 3) and the four-digit level of “Abdominal and Pelvic Pain” (R10) show that 4.7% were coded with “Acute Abdomen” R10.0), 0.5% with “Pain Abdominal, Lower Abdomen Pelvic or Perineal” (R10.2), 38.0% “Pain Abdominal Lower Abdomen” (R10.3) and 41.5% with “Other and Unspecified Abdominal Pain” (R10.4), (Fig. 4).

In the top 30 positions we found another -potentially risky- condition “Phlebitis and Thrombophlebitis” (I80). This could also be evaluated as less serious in terms of the need for medical treatment at the first look. However, some of these conditions were associated with major health problems at the four-digit level and the referral rates to hospital. For example we could observe several cases of thrombosis, phlebitis und thrombophlebitis of the femoral vein (I80.1) or phlebitis and thrombophlebitis of other deep vessels of lower extremities-deep vein thrombosis NOS (I80.2).

## Discussion

After analysing the ICD-10 diagnoses of 163,711 RFEs in Baden-Wuerttemberg for the observation year 2012, from a medical point of view – and without knowledge of social and contextual factors- our analysis point in the direction that there seemed to be numerous RFEs in OOHC that are of minor urgency. Our assessment was based on the rates of referrals to hospital and from the evaluation of the ICD-10 diagnoses at the four-digit level. Overall, we observed referral to hospital rates of less than 5% in OOHC. Other studies showed a referrals to hospitals rate of 9.5% [24, 25]. It could be argued that FPs working in OOHC services in Baden-Wuerttemberg might have higher “tolerance of risk”-scores than in those studies quoted. The low observed rate of referrals to hospitals -with particular focus on the age and gender of the OOHC-patients- in our study points in the direction that evaluated reasons for encounter of the AOK-insured patients of Baden Wuerttemberg were probably less urgent. We could see that male patients and older patients were admitted more often to hospital in accordance with other studies [26]. However, we could not evaluate individual patient characteristics as done by Zwaanswijk et al., who focused on three frequently presented diagnoses in OOHC, to determine the urgency more clearly, because of the plethora of diagnoses in our study [26].

However, we had the chance to stratify for age (<= 50a and >50a) and gender (male/female) and at least we did not see great differences considering the ranking of RFEs.

In our study we found a high proportion of younger people accessing OOHC. It could be assumed that short business hours in regular care (in Baden-Wuerttemberg from 7:00 a.m. to 7:00 p.m. on Monday, Tuesday and Thursday and from 7:00 a.m. to 2:00 p.m. on Wednesday and Friday) may increase the occupation of OOHC for people who work full time. There is evidence, that working individuals make use of OOHC services because of limited time. There is also evidence, that younger patients are more often frequent attenders in OOHC thinking all help request could be handled in OOHC [27, 28].

The assessment of the urgency of reasons for encounter is a central topic and one of the main health services research questions in OOHC. General Practices, Emergency Departments (ED) and OOHC-centres are overcrowded because of millions of attendances. It depends on the definition and of the contextual factors, if consultations are estimated as “Minor Ailments”. In the available literature the percentage of minor ailments varies from 8 to 40% [15, 17, 29–33].

Our finding that “Low Back Pain-Dorsalgia” (M54) - in the broadest sense musculoskeletal pain - was the most frequent diagnosis is consistent with the published results in the available literature. Overall, pain is one of the most common reasons for visiting an ED or OOHC center, but not often a stringent indication to visit an ED or OOHC-center [34–37]. Injuries were found twelve times in the top 30 most frequent diagnoses with low onward referral rates i.e. mostly handled by the FPs in OOHC-centres. Fractures of extremities and severe wounds had to be referred to hospitals. Van der Straten et al. showed similar results in their study [38].

The most frequent infectious disease was the “Acute Upper Respiratory Infection of multiple and unspecified Sites without Complications” (J06). Urinary tract infections were frequent, too, but embedded in the ICD-10 diagnosis “Other Diseases of Urinary System” (N39) and therefore not easy to determine. These findings are nearly congruent with the results of other studies [17, 26, 35, 39–43].

The “Abdominal and Pelvic Pain” (R10) with a 10.6% rate of referrals is one exception in our study regarding the hospitalization rate within the ten most frequent diagnoses in OOHC. It can be supposed that there are involved all the suspected diagnoses like acute appendicitis and acute gynecologic diseases. Here again we have the problem of exact coding. The “Essential (primary) Hypertension” (I10), position eight of the most frequent diagnoses with a 5.6% referrals to hospitals rate, is the second exception. Only 0.7% of the essential primary hypertension diagnoses were coded with “malignant hypertension” (I10.1) in the four-digit levels. This is undoubted a reason for referrals to hospitals. Additional diagnoses like cardiac degeneration or insufficiency possibly lead to the significantly higher referral rates, again a problem of exact coding.

If we want to reduce the high workload in OOHC, we have to give, on the one hand, priority to the high urgency cases and, on the other hand, we have to look at alternatives, such as more telephone advice to patients with minor ailments. Well-established triage systems such as in the Netherlands, Scandinavian countries and the United Kingdom can be helpful to filter the reasons for encounter with regard to urgency with the mentioned constraints of Campbell et al. and Pereira and Wilkie [7, 8, 12–14]. Results from studies, such as ours, can highlight the frequency and urgency of reasons for encounter in OOHC and could be used in education programmes for nurses and physicians working in OOHC and for developing specific guidelines for OOHC in order to support adequate decision making. Such guidelines, may be, could possibly reduce thereby the referral rates of overcautious physicians after hours) [24, 44]. Importantly, OOHC guidelines are not yet available for Germany.

Additionally, we have to strengthen the awareness of patients how to handle symptoms or problems that from a medical point-of-view do not need urgent medical treatment. Education programmes, provided by statutory or private health insurance companies or by community colleges with FP-teachers could help to achieve these goals [27].

One of the strengths of this study is the large sample of solely FP-OOHC data covering a whole federal state of Germany both in rural and urban areas. 5,892 OOHC service centres could be included in this study. We were able to have a closer look on the ICD-10 diagnoses until to the four-digit level and to evaluate these diagnoses by an expert panel of General practitioners (long experience as GPs in own practice) and health services researcher (long experience with health services questions in the field of OOHC). Additionally the assessment of the severity of the diseases was ensured by the referrals to hospitals rate.

In terms of limitations in this study, we acknowledge that patients’ subjective assessments of their diseases and their sociodemographic status have to be considered when discussing urgency or minor ailments in OOHC. “Worry” is a frequent motive of patients presenting at an OOHC centre [20, 21, 27].

Another limitation is the individual, variable and blurred encoding (abdominal pain may indicate an acute appendicitis or constipation for example). We acknowledge that it is difficult for FPs to make an exact diagnosis in the OOHC setting. The precision of the coding is associated with the knowledge of the medical history of the patient and with the course of the suspected disease. It is difficult to implement this essential precondition in any given context. Moreover it was not possible to unambiguously identify the physician on duty. This is the case when the physician on duty is not a resident doctor. In these cases the identifier of the superordinate physician is recorded.

Another limitation is that, in Germany, referrals to specialists in ambulatory health care must not be issued in OOHC. So, this further possibility to estimate the severity of reasons for encounter could not be included. Nevertheless the ICD-10 codes reflect the severity of diseases perhaps better than ICPC-codes in a previous study could do [26]. Finally, we could not take into account the travel distance between the patient’s home and the OOHC-centre. A greater distance is associated with lower utilization of OOHC-centres, whereby distances within Baden-Wuerttemberg are comparatively short, at least in comparison to Norway [45].

## Conclusions

The results of our study suggest that there is a not negligible number of patients in German primary OOHC with comparatively minor ailments. More research resources should be invested in further studies in order to identify and analyse more concisely minor ailments reliable, and to improve the overall progress of OOHC. Additionally, results like these can be used in staff education especially improving triage methods and medical recommendations. Until now, there are no specific guidelines for OOHC in Germany in order to support adequate decision making. These mentioned points should be elaborated in prospective studies and then addressed in training programmes considering patient perspectives of their reasons for encounter and strengthen the awareness of patients with specific education programmes.

## Additional files

Additional file 1: Data (British Language). (XLSX 381 kb)

Additional file 2: Data (German Language). (XLSX 381 kb)

## Abbreviations

AOK: Allgemeine Ortskrankenkasse (German health insurance Company)
ED: Emergency department
FP: Family practitioner
HZV: Hausarztzentrierte Versorgung (family doctor coordinated care)
ICD-10: International Classification of Diseases
OOHC: Out-Of-Hours Care
