# Erratum to: Out of hours care in Germany - High utilization by adult patients with minor ailments?

**DOI:** 10.1186/s12875-017-0632-2

**Published:** 2017-05-12

**Authors:** R. Leutgeb, P. Engeser, S. Berger, J. Szecsenyi, G. Laux

**Affiliations:** University Hospital Heidelberg, Marsilius-Arcades, Western Tower, Im Neuenheimer Feld 130.3, 69120 Heidelberg, Germany

## Erratum

After publication of the original article [1], it came to the authors’ attention that Figs. 2, 3 and 4 were presented incorrectly. Upon investigation, a change requested by the author at proofing had been misinterpreted, resulting in the Figures and their captions being out of sequence.

**Fig. 2:**
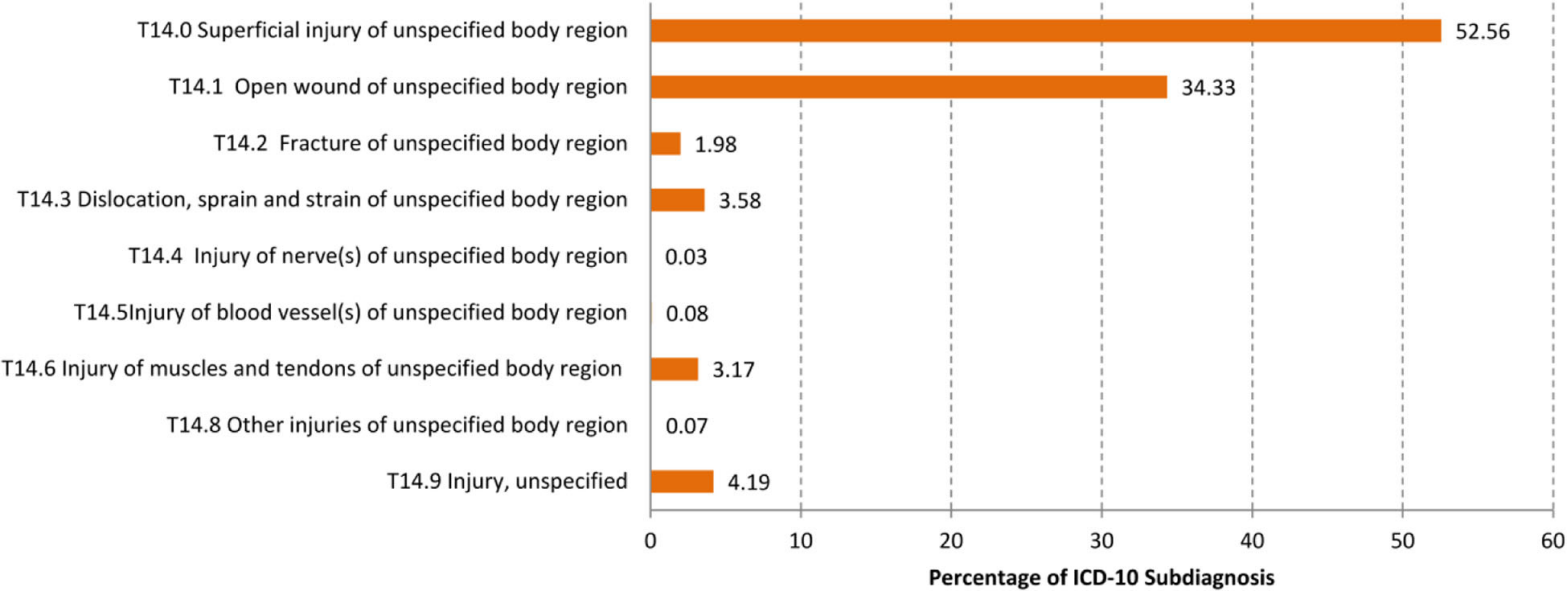
Distribution of the diagnoses “Injury of unspecified body region” (T14) at the four-digit level

**Fig. 3:**
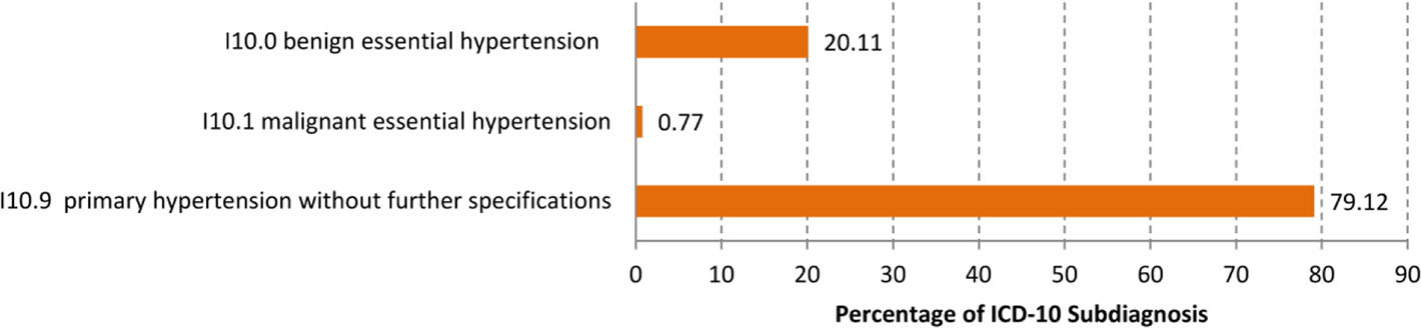
Distribution of the diagnoses “Essential (primary) hypertension” (I10) at the four-digit level

**Fig. 4:**
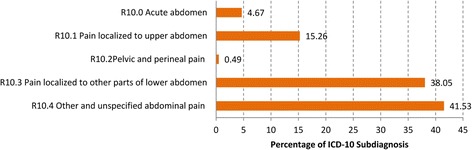
Distribution of the diagnoses “Abdominal and Pelvic Pain” (R10) at the four-digit level

The original article has now been updated in order to rectify the error, and the Figures are published in their correct form in this erratum.
